# The Case for Improved Interprofessional Care: Fatal Analgesic Overdose Secondary to Acute Dental Pain during Pregnancy

**DOI:** 10.1155/2016/7467262

**Published:** 2016-10-26

**Authors:** Sarah K. Y. Lee, Rocio B. Quinonez, Alice Chuang, Stephanie M. Munz, Darya Dabiri

**Affiliations:** ^1^Department of Prosthodontics, School of Dentistry, University of North Carolina at Chapel Hill, Chapel Hill, NC, USA; ^2^Department of Pediatric Dentistry and Pediatrics, Schools of Dentistry and Medicine, University of North Carolina at Chapel Hill, Chapel Hill, NC, USA; ^3^Department of Obstetrics and Gynecology, School of Medicine, University of North Carolina at Chapel Hill, Chapel Hill, NC, USA; ^4^Department of Oral and Maxillofacial Surgery/Hospital Dentistry, School of Dentistry, University of Michigan, Ann Arbor, MI, USA

## Abstract

Prenatal oral health extends beyond the oral cavity, impacting the general well-being of the pregnant patient and her fetus. This case report follows a 19-year-old pregnant female presenting with acute liver failure secondary to acetaminophen overdose for management of dental pain following extensive dental procedures. Through the course of her illness, the patient suffered adverse outcomes including fetal demise, acute kidney injury, spontaneous bacterial peritonitis, and septic shock before eventual death from multiple organ failure. In managing the pregnant patient, healthcare providers, including physicians and dentists, must recognize and optimize the interconnected relationships shared by the health disciplines. An interdisciplinary approach of collaborative and coordinated care, the timing, sequence, and treatment for the pregnant patient can be improved and thereby maximize overall quality of health. Continued efforts toward integrating oral health into general healthcare education through interprofessional education and practice are necessary to enhance the quality of care that will benefit all patients.

## 1. Introduction

The pregnant dental patient exemplifies the need for collaborative practices between health disciplines. The latest national consensus statement regarding oral healthcare during pregnancy indicates patients can and should undergo routine dental treatment during all stages of pregnancy as “oral health care, including use of radiographs, pain medication, and local anesthesia, is safe throughout pregnancy” [1]. While treatment rendered during the second trimester provides the greatest comfort, pregnancy alone is not a contraindication to receiving dental treatment [1]. For some women, pregnancy may in fact provide the opportunity to pursue their oral healthcare needs [2, 3]. For example, some states' government assistance programs include dental care as a covered pregnancy-related service [4]. In 2000, the Children's Health Insurance Program extended coverage to include pregnant women who do not qualify for Medicaid [3].

Despite these progressive efforts to provide prenatal oral healthcare, inconsistencies between the knowledge and practices of dental and medical providers regarding prenatal oral healthcare remain. Pregnant patients continue to encounter barriers that may adversely affect their oral health and negatively impact their pregnancy [1–11]. This case report describes a sequence of events, precipitated by dental pain, in which lapses in patient oral health literacy, the rendering of dental treatment, and coordination of interprofessional collaborative treatment within the healthcare system culminated in the demise of both the fetus and pregnant patient.

## 2. Case Presentation

A 19-year-old at 17-week gestation presented to her local hospital's emergency department (ED) complaining of abdominal pain and nausea. She was diagnosed with acute liver failure secondary to acetaminophen overdose for dental pain management. The admission record indicated, as per patient report, that she had received dental treatment 2 weeks earlier, with the dentist reportedly prescribing 20 tablets of Tylenol #3 (acetaminophen with codeine) for postoperative pain. The patient initially took 1-2 tablets per day, but due to persisting symptoms, she communicated with her obstetrician who recommended over-the-counter Tylenol for pain management. The patient obtained Extra Strength Tylenol (500 mg acetaminophen/tablet) and for a 10-day period reported taking 2-3 tablets of Extra Strength Tylenol, 10 times per day, approximating 20–30 tablets daily or 10,000–15,000 mg daily. Preliminary ED laboratory studies indicated acute liver injury consisting of coagulopathy and abnormal transaminases with significantly elevated acetaminophen levels. To address the liver toxicity, N-acetylcysteine (NAC) protocol was initiated at the local ED and continued when the patient was transferred to a larger academic center's pediatric intensive care unit (ICU) (Table 1).

**Figure 1:**
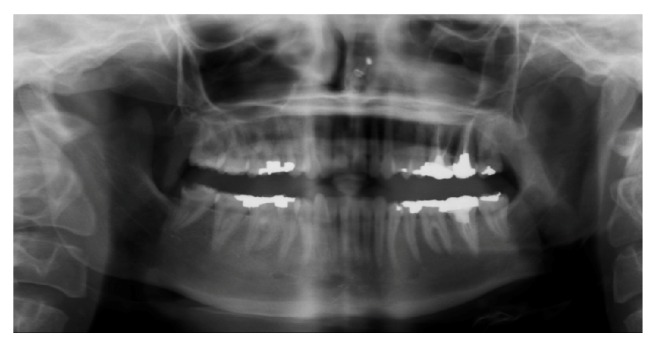
Panoramic radiograph of the patient's dental condition on day 15 of first hospitalization.

When transferred, a consultation with obstetrics and gynecology (ObGyn) was completed. A live singleton fetus had been initially confirmed by ultrasound; however, on reevaluation on her second day of hospitalization, no fetal cardiac activity was detected and fetal demise was diagnosed. The following day, a dental consultation was initiated due to the patient's complaint of pain on mastication of the right mandibular dentition. Clinical and radiographic examination initially revealed no emergent dental needs, and occlusal adjustments to alleviate symptoms were performed as the first course of action (Figure 1).

While undergoing care the patient was diagnosed with Wilson's disease, an autosomal recessive genetic disorder causing copper accumulation in tissues that can lead to further liver complications [12]. Her laboratory findings confirmed the abnormally elevated copper levels, which in addition to her acute liver injury from toxicity resulted in the recommendation for liver transplant. On day 9 of hospitalization, the delivery of the nonviable fetus was completed, and the patient's condition was reported as stable. At this time, a second dental consultation was ordered following the patient's report of a “bubble on [the] gum that popped.”

The dental assessment revealed that tooth #30 (permanent right mandibular first molar) had a draining sinus tract. Two days following the diagnosis, prophylactic antibiotic management was initiated and a pulpectomy was scheduled and completed in the hospital's dental clinic under local anesthesia. On the scheduled treatment date, the patient reported not feeling well as she had not ingested solid food or substantial liquids for more than 12 hours, due to her* nil per os* (NPO) status as ordered by her medical care team. While this action resulted in delay of treatment, the pulpectomy was completed without complication that same afternoon. At this time, the dental team overseeing the patient's care discussed the previously rendered treatment with the patient's general dentist via telephone. The following dental treatment had been reportedly completed in a single appointment by the general dentist and was documented in the patient's electronic record: dental restorations on 7 teeth (#12, 13, 16, 17, 19, 20, and 21), 3 root canal therapies (#14, 15, and 18), and placement of 2 stainless steel crowns. Further requests were made to the dentist to share treatment records with the hospital dentistry team. To date, these records have not been received.

Dental clearance evaluation and any necessary treatment in preparation for a liver transplant were requested by the patient's medical team. The following treatment was then recommended: endodontic therapy of pulpal necrosis with sinus tract of tooth #30, extraction of tooth #14 (permanent left maxillary molar) due to nonrestorability, and extraction of maxillary and mandibular third molars (teeth #1, 16, 17, and 32) due to impaction causing operculi and periodontal complications. The patient was subsequently discharged from the hospital with plans to address dental treatment needs and management of liver failure by the respective care teams on an outpatient basis.

One week after discharge, the patient was readmitted to the ED for pelvic pain that had been worsening for 3 days. She disclosed, as documented on her electronic record, a lack of compliance with the prescribed medication regimen “as she does not know what these medications do.” Treatment for spontaneous bacterial peritonitis (SBP) was initiated by the gastroenterology (GE) team but was discontinued after 2 days due to lack of correlation of signs and symptoms observed from laboratory studies and patient history. During this stay, a dental follow-up evaluation was completed. The patient reported being asymptomatic for any oral pain and, upon clinical examination, the draining sinus tract adjacent to tooth #30 had resolved. No emergent needs were evident. The patient was discharged after a 4-day hospitalization.

Three days after her second hospitalization, the patient presented to the dental outpatient clinic for completion of endodontic therapy for tooth #30 and consultation with the oral and maxillofacial surgery (OMFS) service for extractions of third molars and tooth #14 under general anesthesia. Endodontic therapy was completed in the dental clinic without complication and the patient was scheduled to return to complete other indicated restorative treatments. The extractions were completed in the operating room under general anesthesia by the OMFS service the same week without complications.

Three days following the extractions, the patient reported a three-day history of abdominal pain, nausea, and diarrhea. Laboratory studies indicated evidence of leukocytosis, resulting in admission to the hospital's adult ICU. The patient was newly diagnosed with portal hypertensive gastropathy with ascites, due to portal hypertension with SBP. Complications led to degradation of her condition requiring intubation and broad-spectrum antibiotic therapy. After one week, her condition stabilized and she was extubated. Paracentesis was completed to remove 4 liters of fluid. An isolated episode of unresponsiveness to sternal rubbing occurred. This was managed by administration of 1.2 mg IV Narcan and later attributed to Phenergan sedation. After 2.5-week hospitalization, the patient was discharged.

The following week, the patient was found to be unresponsive at her home and was readmitted to the ICU. She was observed to be significantly obtunded and jaundiced with distended abdomen and remained minimally responsive. She was eventually diagnosed with septic shock secondary to SBP with new onset of acute kidney injury and hypotension. The option to use continuous renal replacement therapy was declined by the patient's family. On day 2 of her ICU stay, cardiac ischemia was evident with development of nonsustained ventricular tachycardia. She developed intermittent prolonged unstable arrhythmia with ventricular fibrillation and hypotension. The maximum amount of norepinephrine was administered to counter hypotension and heart failure. After the care team discussed the patient's poor prognosis, her family decided to halt further life-sustaining measures. After a 3-day ICU course, the patient died.

## 3. Discussion

Consolidated guidelines have been established by both separate and collaborative medical and dental organizations to foster and support care integration, particularly for pregnant patients [1, 5]. Provision of prenatal oral healthcare must be managed in a safe and appropriate manner. The quantity of rendered treatment and the postoperative complications prompted the initiation of this patient's course of illness and may have contributed to exacerbation of liver symptoms in conjunction with her unknown, preexisting condition of Wilson's disease. Dentists provide expertise and means for diagnosing, planning, treating, and educating the patient to optimize oral health. With the provision of dental care, the health risks and benefits of providing an extensive amount of invasive treatment, regardless of the patient's pregnancy status, must be considered. For the pregnant patient, it is critical to assess the impact of dental treatment during pregnancy in terms of priority (emergent versus routine), quantity, timeliness, medications involved in rendering treatment, ergonomics while undergoing treatment, and management of posttreatment complications, including pain. It is especially critical that the pregnant patient obtains treatment when she presents with an acute odontogenic infection, as delays can carry greater risks than those associated with exposure to treatment and medications required for management. The use of local anesthetic, modalities of sedation, and analgesia in the pregnant patient has been complex and controversial [9]. National consensus statements and recent studies have rendered many of these modalities safe when used properly, in consultation with the prenatal provider when needed [1, 5, 9, 13].

Medical providers also contribute expertise to the pregnant patient's oral healthcare. Just as dental providers must take the modifications and potential complications associated with rendering dental care of any patient into consideration, medical providers must also address the oral health needs that may arise in their own patient management. As a provider who also consistently manages the patient throughout her pregnancy, the medical provider is able to identify the need for the patient to be referred for dental care [1, 5, 7, 8, 10]. The medical provider serves as a source of disseminated information that may encourage prevention and early intervention of oral health problems such that these problems and their consequences can be better managed [1, 5]. This provider can also communicate with the dental provider on systemic health considerations such that care can be rendered safely [1, 5].

Evidence-based dental management of the pregnant patient continues to be practiced inconsistently [1, 7, 8, 10, 11, 14–16]. Many pregnant patients are still unable to find a dental provider willing to treat them due to remaining misconceptions regarding oral healthcare. Concerns of unfounded risks to the fetus with dental treatment heighten issues of premature induction labor, lack of knowledge in the safety of treatment, and potential legal risks if negative birth outcomes occur [4, 6, 8, 10, 11]. These are commonly perceived deterrents [8, 10]. Limitations based on incorrect or insufficient knowledge of perinatal oral healthcare by the treating dentist have been shown to have the strongest direct effect on preventing pregnant patients from obtaining dental care [11]. Dentist-imposed barriers to accessing reasonable care can lead to deleterious effects and create greater risk management issues.

Medical professionals have similar hesitations when addressing their pregnant patients' dental status. General health practitioners, midwives, and obstetricians reported their lack of knowledge in understanding the safety of prenatal dental treatment as the most significant limitation [10]. These providers also reported feeling unqualified to address dental issues due to insufficient familiarity and knowledge on oral health topics [8], highlighting the importance of proper training and the need to address these topics in medical and dental curricula. Many educational institutions among the health disciplines exhibit organizational infrastructure, logistical barriers, and isolated education that continue to support a discord at odds with current recommendations [13–15, 17].

While each profession maintains management practices specific to its discipline, it is significant to acknowledge the interrelatedness of the health professions and how care coordination impacts the pregnant patient's overall health. Pregnancy, as a sensitive period in which compromises in oral-systemic health can readily occur, typifies the importance of establishing and maintaining coordination between medicine and dentistry as well as other healthcare professions. Adverse outcomes occur as a result of discordant care among the health disciplines. Pregnant patients and their fetuses are placed at greater risks when preventive and intervening therapies are not provided in a timely and appropriate manner [1, 4]. In this case, for example, the patient's unnecessary placement on NPO status revealed a misunderstanding and lack of communication between the teams coordinating her care. As a result, the patient's treatment was delayed due to her poor disposition. Additionally, missed opportunities for collaboration reinforced the separation of health disciplines and the notion of integrated general health in the mindsets of both providers and patients.

It is important to recognize that while healthcare providers carry many responsibilities in managing a patient's health, the patient is also an active participant in the outcomes that emerge from care. Acetaminophen, a perinatal-appropriate pain medication with a recommended maximum dosage of 4 g in a 24-hour period [18], was independently prescribed by her medical and dental providers, but misused by the patient. This misuse led to an overdose that precipitated the adverse chain of events. As a first-time pregnant, low-income adolescent, this patient belonged to a population that is more susceptible to adverse health outcomes resulting from low health literacy, defined as the “degree to which people have the capacity to obtain, process, and understand basic health information and services that are needed to make appropriate health decisions” [6, 7]. Low health literacy has been associated with poorer health knowledge that can be attributed to poorer health behaviors and outcomes [6, 7, 19]. While undergoing care to manage complications that resulted from the overdose, this patient exhibited noncompliant behavior that further compromised treatment. These key instances reflect a misunderstanding and misuse on the patient's part of the information and resources available to her.

Health literacy is not wholly dictated by the patient's individual characteristics such as socioeconomic status and level of education; the degree of literacy is influenced by established systems of communication for information dissemination and patient education [19, 20]. Social and cultural misconceptions about undergoing care during pregnancy [13] and the lack of awareness of their oral health status and its impact on their pregnancy and general health [4, 8] are contributing barriers that prevent patients from accessing and utilizing care.

Healthcare entails the overall management of the well-being of a patient in aspects of education, treatment, and maintenance. Historically, dentistry has been a very separate branch of healthcare [13–17, 21], practiced on different educational infrastructure, clinical management, and financial models more than medicine [16]. Unfortunately, the disparities in care that have resulted from persisting separation of disciplines are still evident in modern day healthcare practices.

Efforts to integrate dentistry with other health professions have increased with recognition of oral health implications in general health by the medical community, development of collaborative medical-dental training, and incorporation of oral health in medical settings. Unfortunately these efforts remain limited in the educational arena, in part because of the segmented and isolated educational systems between the health branches that have fostered gaps in knowledge and clinical practice between oral and general health [22, 23].

A survey of US dental school indicated a willingness by educators to incorporate prenatal oral health, but clinical experiences remain limited. Barriers included lack of pregnant patients and faculty expertise [24]. Similarly in Canadian dental schools, only 40% of schools report having designated time in their curriculum to cover this topic [25]. Initiatives such as the Prenatal Oral Health Program (pOHP) at the University of North Carolina show promise in helping educate the next generation of providers in a collaborative approach in practice and thereby, improve the quality of rendered care and patient outcomes [26, 27].

Standardization of coordinated care within clinical and educational institutions is likely to be a prolonged process where results may not be rapidly realized. Attitudinal and behavioral practice changes of dental providers to address the needs of high risk populations for adverse health outcomes, as well as prioritizing collaborative efforts with healthcare colleagues and educational initiatives across health professional schools, are essential for tangible, meaningful progress in oral health disparities to occur.

## 4. Conclusion

This case highlights how practice misconceptions, barriers in collaborative care and communication, and insufficient health literacy are interconnected and complicated by one another. Though specific to the pregnant dental patient, this case offers lessons that can be readily translated to any type of patient, especially other susceptible populations, including the frail elderly, patients with medical complexity, and those with disabilities. Oral health is one part that contributes to overall health. As such, it is important to recognize that a patient's well-being relies on the coordinated efforts of all the health disciplines. This case report highlights some of the challenges of incorporating dental and medical practices within the current healthcare environment. Most prevalent of these issues were the dental and medical providers' inconsistencies in patient management, the segmented, noncollaborative infrastructure of communication and care coordination between these providers, and the patient's lack of knowledge and understanding of her health status. The coordinated efforts between specialties made in the latter part of this patient's care are evidence that collaboration, albeit challenging, is readily possible and critically necessary. Greater emphases on interprofessional education, practice, and systems changes are needed to help address some of the current clinical challenges and disconnects among healthcare professions.

## Figures and Tables

**Table 1:** Summary of the patient's course of illness.

	Date	Event
2 weeks before hospitalization		Patient obtains dental care. Her dental provider prescribed 20 tabs of Tylenol 3 for pain management. Patient takes 1-2 tabs/day but pain persists. She contacts her obstetrician who advises OTC acetaminophen for pain management. Patient obtains Extra Strength Tylenol (500 mg acetaminophen/tab) and takes 2-3 tabs, 10 times/day for last 10 days (20–30 tabs/day).

First hospitalization	Day 1	Patient presents to local emergency department (ED) for abdominal pain and nausea. Diagnosis of acute liver injury is assessed. N-Acetylcysteine (NAC) treatment is initiated.
	Day 2	Patient transferred to pediatric ICU and liver management continued via NAC protocol. Obstetrics and gynecology (ObGyn) team identifies live singleton fetus via ultrasound.
	Day 3	Undetectable fetal heart sounds or fetal movement by ObGyn. Fetal demise diagnosis is made.
	Day 4	Patient reports pain on mastication of right posterior dentition. Hospital dentistry (HD) consult is requested by patient's care team.
	Day 5	HD consultation completed. Clinical examination reveals no indication for emergent interventional dental treatment. Palliative treatment is rendered via occlusal adjustment.
	Day 7	Wilson's disease diagnosis made. The patient continues undergoing management of acute liver injury.
	Day 10	Delivery of nonviable fetus performed.
	Day 13	Patient reports “bubble on gum that popped” but is asymptomatic. HD consultation completed and reveals tooth #30 (right mandibular molar) has draining sinus tract.
	Day 15	Pulpectomy performed on tooth #30 in hospital dental clinic. Dental needs for liver transplant clearance are assessed and scheduled for treatment on an outpatient basis. Patient's original dentist is contacted via telephone and relays that patient received dental treatment of 10 left posterior teeth, including root canal therapy on 3 molars in 1 visit. Patient discharged from hospital.

*6 days after hospitalization*		
Second hospitalization	Day 1	Patient presents to ED for worsening and persistent pelvic pain. Patient admitted for management with Gastroenterology (GE) team and spontaneous bacterial peritonitis (SBP) treatment is initiated and paracentesis completed.
	Day 2	HD consultation for post-pulpectomy follow-up. Patient's dental condition is stable and patient is scheduled for further dental management on an outpatient basis.
	Day 3	SBP treatment discontinued.
	Day 5	Patient discharged from hospital.

13 days after hospitalization	Day 3	Oral maxillofacial surgery (OMFS) consultation is completed for extractions under general anesthesia.
	Day 6	OMFS completes dental treatment under general anesthesia (teeth 1, 14, 16, 17, and 32 extracted).

Third hospitalization	Day 1	Patient presents to ED for abdominal pain, nausea, and diarrhea. Patient admitted to adult ICU.
	Day 2	Patient's condition deteriorates and patient is intubated.
	Day 3	Patient diagnosed with portal hypertensive gastropathy and ascites. Continued management of liver complications including paracentesis and esophagogastroduodenoscopy (diagnostic endoscopic procedure for visualization of upper portion of GI tract).
	Day 5	Patient's condition stabilizes and patient is extubated.
	Day 9	Paracentesis completed with 4 L of fluid removal.
	Day 10	Patient has an episode of unresponsiveness to sternal rub, requiring 1.2 IV Narcan administration before patient's mental status returns. This incident is attributed to Phenergan sedation.
	Day 17	Patient discharged from hospital.

*6 days after hospitalization*		
Fourth hospitalization	Day 1	Patient admitted to local ED after found unresponsive at home. Patient transferred to adult ICU and is intubated. Patient diagnosed for septic shock secondary to SBP.
	Day 3	Patient diagnosed for cardiac ischemia with development of nonsustained ventricular tachycardia. Multiorgan failure is observed. Patient shows intermittent prolonged unstable arrhythmia with ventricular fibrillation and hypotension. Patient's care team discusses poor prognosis with family.
	Day 4	Patient dies.

## Abbreviations

ED: Emergency department
NAC: N-Acetylcysteine
ICU: Intensive care unit
ObGyn: Obstetrics and gynecology
NPO: Nil per os
SBP: Spontaneous bacterial peritonitis
GE: Gastroenterology
OMFS: Oral and maxillofacial surgery.
